# Relationship between visual prostate score (VPSS) and maximum flow rate (Q_max_) in men with urinary tract symptoms

**DOI:** 10.1590/S1677-5538.IBJU.2015.0032

**Published:** 2016

**Authors:** Mazhar A. Memon, M. Hammad Ather

**Affiliations:** 1 Aga Khan University, Surgery karachi, Sind, Pakistan

**Keywords:** IPSS, Prostate, Urinary Tract, Lower Urinary Tract Symptoms

## Abstract

**Objective:**

To evaluate correlation between visual prostate score (VPSS) and maximum flow rate (Q_max_) in men with lower urinary tract symptoms.

**Material and Methods:**

This is a cross sectional study conducted at a university Hospital. Sixty-seven adult male patients>50 years of age were enrolled in the study after signing an informed consent. Q_max_ and voided volume recorded at uroflowmetry graph and at the same time VPSS were assessed. The education level was assessed in various defined groups. Pearson correlation coefficient was computed for VPSS and Q_max_.

**Results:**

Mean age was 66.1±10.1 years (median 68). The mean voided volume on uroflowmetry was 268±160mL (median 208) and the mean Q_max_ was 9.6±4.96mLs/sec (median 9.0). The mean VPSS score was 11.4±2.72 (11.0). In the univariate linear regression analysis there was strong negative (Pearson's) correlation between VPSS and Q_max_ (r=848, p<0.001). In the multiple linear regression analyses there was a significant correlation between VPSS and Q_max_ (β-http://www.blogapaixonadosporviagens.com.br/p/caribe.html after adjusting the effect of age, voided volume (V.V) and level of education. Multiple linear regression analysis done for independent variables and results showed that there was no significant correlation between the VPSS and independent factors including age (p=0.27), LOE (p=0.941) and V.V (p=0.082).

**Conclusion:**

There is a significant negative correlation between VPSS and Q_max_. The VPSS can be used in lieu of IPSS score. Men even with limited educational background can complete VPSS without assistance.

## INTRODUCTION

Lower Urinary Tract Symptoms (LUTS) are common among both aging men and women (1, 2). In several communities based studies it has been reported that LUTS are more common among individuals more than 50 years of age. LUTS are now recognized to be multifactorial. They may be related to body habitus dietary intake, fluid intake and alcohol consumption, age related and from various cardiovascular pathologies. In addition, some medications can also contribute to LUTS (3, 4). In multiple public health studies it has been identified that LUTS have significant implication on quality of life and in severe state they may lead to psychological sequel that may result in depression and anxiety (5, 6). Objective evaluation of LUTS is not only necessary to follow on progression but also to assess the efficacy of treatment (7). Of the many questionnaires to evaluate male LUTS, IPSS is the most preferred one (8, 9).

One of the most significant causes of male LUTS is obstruction; uroflowmetry (UFM) is a simple and non-invasive tool to assess obstruction. UFM determines volume of urine passed per unit of time. It has numeric and graphic representation, which evaluates multiple parameters out of which Voided Volume (VV) and Maximum Flow Rate (Q_max_), the most important (10). It has been observed that 30-70% of men can not complete the IPSS because the questions are difficult to understand as they are wordy and people with lower level of education can not complete it (11, 12). In order to obviate these difficulties, Visual Prostrate Symptoms Score (VPSS) has been introduced. VPSS is a pictorial assessment version of IPSS (1). There is dearth of data on the utility of VPSS in men presumed to have benign prostatic obstruction (BPO). The current study aims to study the relationship between VPSS and maximum flow rate (Q_max_) in men with lower urinary tract symptoms related to BPO.

## MATERIALS AND METHODS

The study was conducted at a tertiary care hospital in a developing country from March 2013 to July 2013. Prior approval was obtained from Institutional Ethical Review board for the study. The prospective approach was taken and cross-sectional study design was used. In order to recruit participants, non-probability sampling i.e. consecutive sampling strategy was used. Sample size of 67 was determined using r=0.36 and 1-α=95%. Inclusion and exclusion criteria were pre-determined. Participants who were male and were more than 50 years old with voided volume of>150mL were included. Participants who were unable to fill out the form due to mental or psychological disturbances, patients who refused to participate, patients who could not undergo UFM and patients who experienced other causes including neurogenic bladder or meatal stenosis were excluded from the study.

**Figure 1:**
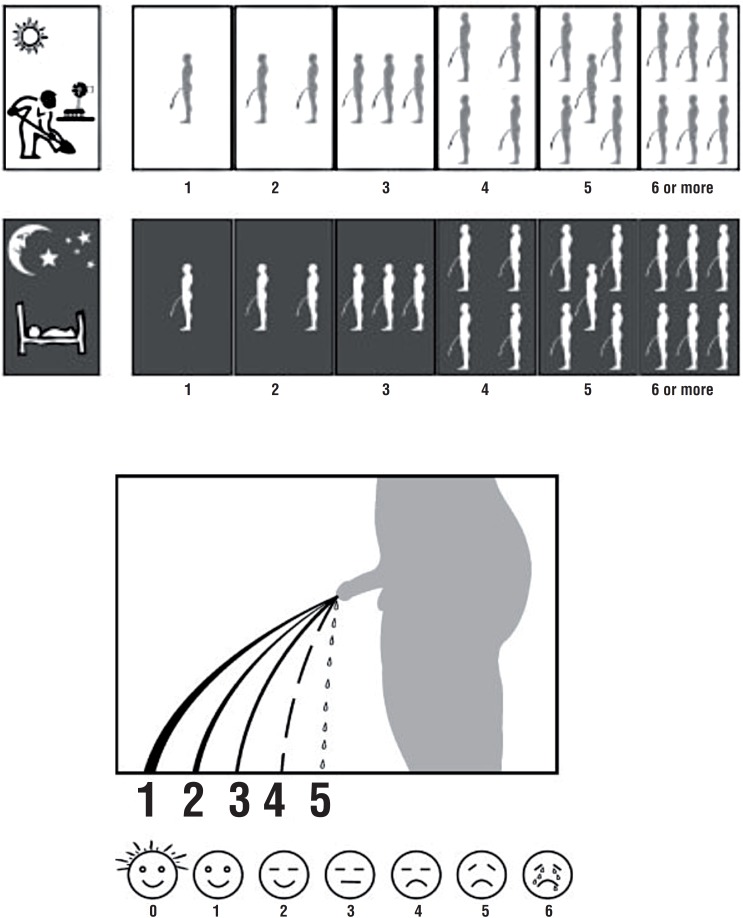
Pictorial display of the visual IPSS (VPSS score), assessing daytime and nighttime frequency, flow rate and QoL.

The data collected included demographic information of participants. In addition, it included VPSS (Figure-1) (http://www.einj.org/journal/view.php?number=147), Q_max_, and voided volume. Level of education was operationally categorized into five categories that included Primary (Grade 1 to 5), Middle (Grade 6 to 8), High (Grade 9 and 10 leading to Secondary School Certificate), Intermediate (Grade 11 and 12 leading to Higher Secondary School Certificate), and University programs leading to undergraduate and graduate degrees. Data was entered and analyzed using SPSS version 19. Mean, median, and standard deviation was computed for continuous variables. Percentages and proportion was computed for categorical variables. Pearson correlation coefficient was computed for VPSS and Q_max_. Moreover, multiple linear relationships were computed to identify relationship between VPSS and Q_max_.

## RESULTS

In total, 67 participants were recruited. The age of study participants ranged from 66–68±10 years. Around 48% of the study participants were not educated, whereas 22% were up to high grade and 30% were Intermediate and above. The mean Q_max_ was 9.69±4.94mL/sec. The mean voided volume recorded for study participants was 268.45±160.49mL. Moreover, the mean VPSS recorded was 11.46±2.72 (Table-1).

**Table 1:** Descriptive Analysis of Study Variables.

Variable	Mean	Median	Standard Deviation
Maximum Flow Rate (Q_max_)	9.69	9.00	4.94
Voided Volume (VV)	268.45	208.00	160.49
Visual Prostrate Symptoms Score (VPSS)	11.46	11.00	2.72

In order to identify the relationship between VPSS and Q_max_, Pearson correlation revealed strong negative relationship (r=-0.848, P<0.001) among these variables. The univariate linear regression analysis revealed that both Q_max_ (P<0.001) and VV (P=0.036) were significantly associated with VPSS. Moreover, multivariate regression analysis revealed significant relationship (P <0.001) between VPSS and Q_max_ while adjusting for the effect of age, VV and level of education (Table-2).

**Table 2:** Univariate and Multivariate Linear Regression Analysis.

Variable		Odds Ratio	Confidence Interval (95%)	P-value
Univariate Linear Regression				
	Maximum Flow Rate (Q_max_)	−0.47	−0.54, −0.39	<0.001
	Voided Volume (VV)	−0.004	−0.008, 0.00	0.036
Multivariate Linear Regression				
	Maximum Flow Rate (Q_max_)	−0.46	−0.54, −0.39	<0.001*

* Adjusting for the effect of age, VV and level of education

## DISCUSSION

The idea of developing VPSS was generated by Van der Walt who identified that patients who were either less educated or not educated required assistance of physicians in order to fill the International Prostrate Symptom Score (IPSS). In comparison, patients were able to comprehend pictorial representation shown in VPSS (13).

Assessment of LUTS is essential to identify and plan management. There are a number of tools available to evaluate LUTS. Among all tools, VPSS has proved to be an effective tool especially for less educated or not educated individuals. It has proved to be an effective tool to assist medical practitioners in making treatment related decisions. In the current study half of the participants were not educated (14, 15).

In line with the findings of the current study, Van der Walt et al. also identified that VPSS correlated with Q_max_. They recruited 96 participants over the period of one year. The identified relationship was negative in nature. They also supported that VPSS is a reliable tool for assessing subjective symptoms of patients experiencing LUTS (13). Similarly, other studies have also reported significant association between VPSS and Q_max_. In our study, the total number of patients was 67 and it was a six months study whereas in previous studies the time period was at least of one year. Yeon Won Park et al. have the highest participant number of 240 and our study was done only during six months (16). More recently Afriansyah and colleagues (17) similarly noted in rural Indonesian areas that VPSS correlated significantly with the IPSS and could be completed without assistance by a greater proportion of men with low levels of education.

It has been proven that patients defined their symptoms accurately proving the strength of VPSS scoring system. Moreover, this can also be interpreted that the patient understands pictogram of VPSS in a proper manner. The actual VPSS score is 23 but as we have excluded the factor quality of life, the total VPSS analysis was 17 instead of 23 and the reason for exclusion is defined above. The comparative study by Van der Walt et al. has weighed all components of VPSS but the drawback of that study is that they have not mentioned mean VPSS score. In our study mean VPSS was 11.4±2.72 and it is a valuable Figure for interpretation. Whereas in another study conducted by Yeon Won Park the mean VPSS was 9, which is slightly lower when compared with our study, but comparable with other study conducted by Heyns et al. (14). In our study the mean maximum flow rate (Q_max_) was 9.69mL, which was slightly lower compared to other studies. About 50 percent of our population was less educated and it was almost comparable to the other studied.

The findings of our study also revealed that age, voided volume and level of education are independent variables. After the multivariate analysis with VPSS we found that there was no significant difference between each other and the calculated P-value for age was 0.460, VV was 0.151 and for LOE P=0.999. Similarly, the study by Heyns et al. (14) also identified that there is no significant association between age and VPSS. In multivariate analysis, when Q_max_ was correlated to VPSS it was observed a significant p value (<0.0001). In our study there was strong negative correlation (−0.848) between VPSS and Q_max_ and when compared to the other studies it was more negative. Although IPSS and VPSS are predominantly used in the evaluation of male LUTS secondary to BPO, Wessel and Heynes (18) noted that they could be effectively used for urethral stricture related LUTS. They noted that VPSS correlates significantly with IPSS, Q_max_, and urethral diameter in men with urethral stricture disease and takes significantly less time to complete. A combination of VPSS>8 and Q_max_<15mL/s can be used to avoid further invasive evaluation during follow-up in men with urethral strictures.

Our study proved that level of education doesn't have significant association with VPSS. This supports that less educated or uneducated patient can provide better conclusion when they filled these pictorial form of VPSS, which is used for objective evidence for LUTS. The main drawback of our study is that we have not compared the VPSS with IPSS and also with other parameters of uroflowmetry.

## CONCLUSIONS

The current study has shown a significant negative correlation between VPSS and Q_max_. VPSS is a pictorial form rather than descriptive and can be completed without assistance by men with limited education.
